# Mammalian cell growth on gold nanoparticle-decorated substrates is influenced by the nanoparticle coating

**DOI:** 10.3762/bjnano.5.257

**Published:** 2014-12-24

**Authors:** Christina Rosman, Sebastien Pierrat, Marco Tarantola, David Schneider, Eva Sunnick, Andreas Janshoff, Carsten Sönnichsen

**Affiliations:** 1 Institute of Physical Chemistry, University of Mainz, Duesbergweg 10–14, 55128 Mainz, Germany; 2 Fraunhofer Institute for Microelectronic Circuits and Systems (IMS), Finkenstraße 61, 47057 Duisburg, Germany; 3 Laboratory for Fluid Dynamics, Pattern Formation and Biocomplexity, Max Planck Institute for Dynamics and Self-Organization (MPIDS), Am Fassberg 17, 37077 Göttingen, Germany; 4 Institute of Physical Chemistry, University of Göttingen, Tammannstraße 6, 37077 Göttingen, Germany,; 5 Max Planck Institute for Biology of Ageing, Joseph-Stelzmann-Straße 9b, 50931 Cologne, Germany

**Keywords:** basolateral application, cytotoxicity, electric cell–substrate impedance sensing, gold, nanoparticles

## Abstract

In this work, we study epithelial cell growth on substrates decorated with gold nanorods that are functionalized either with a positively charged cytotoxic surfactant or with a biocompatible polymer exhibiting one of two different end groups, resulting in a neutral or negative surface charge of the particle. Upon observation of cell growth for three days by live cell imaging using optical dark field microscopy, it was found that all particles supported cell adhesion while no directed cell migration and no significant particle internalization occurred. Concerning cell adhesion and spreading as compared to cell growth on bare substrates after 3 days of incubation, a reduction by 45% and 95%, respectively, for the surfactant particle coating was observed, whereas the amino-terminated polymer induced a reduction by 30% and 40%, respectively, which is absent for the carboxy-terminated polymer. Furthermore, interface-sensitive impedance spectroscopy (electric cell–substrate impedance sensing, ECIS) was employed in order to investigate the micromotility of cells added to substrates decorated with various amounts of surfactant-coated particles. A surface density of 65 particles/µm^2^ (which corresponds to 0.5% of surface coverage with nanoparticles) diminishes micromotion by 25% as compared to bare substrates after 35 hours of incubation. We conclude that the surface coating of the gold nanorods, which were applied to the basolateral side of the cells, has a recognizable influence on the growth behavior and thus the coating should be carefully selected for biomedical applications of nanoparticles.

## Introduction

Over the last decade, the biomedical applications for gold nanoparticles have become increasingly diverse due to their small size and plasmonic nature [1]. The plasmon resonance wavelength of the nanoparticle, which exhibits strong light scattering and absorption, can be controlled by synthesis conditions [2] in order to match the “optical window” of biological tissue in the wavelength region of 650–900 nm [3]. Therefore, gold nanoparticles can be used, for example, as biosensors [4–5], as delivery systems [6–7], as contrast agents in imaging [8–9], and as tools for photothermal therapy [2,10]. However, the impact of functionalized nanomaterials on living organisms is still not fully understood and the number of studies on this topic are few compared to the number of nanoparticle types and applications [11].

To date, the studies are focused on nanoparticle application from the apical side of the cells [12]. Here, adherent cells are grown to various degrees of confluence, the nanoparticles are applied suspended in cell medium, and finally the uptake and/or the influence on metabolic activity is quantified. Quantification of uptake numbers occurs by microscopic and spectrometric methods, whereas the metabolic activity is assessed by various biochemical assays.

In contrast, cell growth on nanoparticle-decorated surfaces mimicking basolateral nanoparticle application is only weakly characterized so far, although a basolateral interaction is possible after nanoparticle infiltration into a tissue lesion or by insertion of nanoparticle patterned implants. This can potentially influence cell migration, which has implications in wound healing [13]. Recently, a study by Yang et al. tracked the migration behavior of prostate carcinoma cells (PC3, epithelial) and human dermal fibroblast cells (HDF) on gold nanoparticle-patterned surfaces for almost 10 h using optical dark field microscopy [14]. The authors found that the sedimented nanoparticles were collected by the cells during movement, which is clearly seen by a trail free of particles left behind. This property (i.e., the marking of cell movement by the voids created on a nanoparticle carpet) was used already in 1977 to visualize cell migration [15].

Because nanoparticles are so prevalently used to coat surfaces (for instance, to create biofilm resistance on implants [16], to enhance stability or to create a special functionality), this study is focused on the impact of basolateral exposure of gold nanoparticles on epithelial cells. Here, epithelial cells were exposed to nanoparticles adsorbed onto a surface. Since MDCK II cells exhibit caveolae only basolaterally, it is conceivable that internalization is enhanced compared to apical exposure [17]. In this study, epithelial cells grown on substrates decorated with gold nanorods exhibiting different surface coatings are compared with cells growing on bare substrates in order to assess the impact of the coating agents on basolateral nanoparticle application. Live cell imaging was performed over the course of an incubation time of three days using optical dark field microscopy in order to evaluate the cell adhesion and spreading by the cell morphology. We observe an influence of the particle coating on the growth behavior with respect to the cytotoxic properties of the coating agent and its reactive group. The impact on surfactant-induced cell behavior was investigated in more detail by interface-sensitive impedance spectroscopy (electric cell–substrate impedance sensing, ECIS). Studies on the uptake and influence on metabolic activity with respect to apical application of the same functionalized nanoparticles are presented elsewhere [18–20].

## Results

### System

We investigate the growth of mammalian epithelial kidney (MDCK II) cells seeded on glass substrates decorated with gold nanorods (38 × 17 nm, Supporting Information of [20]) and compare the results to cell growth on bare substrates. The study utilized three different surface coatings because the particle-bound molecules (stabilizing agents) are expected to promote diverse interactions with the cell membrane [21–22]. One coating consists of cetyltrimethylammonium bromide (CTAB), which is a relatively cytotoxic cationic surfactant [11] present on the particle surface after synthesis. These CTAB molecules can be replaced by the inert polymer poly(ethylene glycol) (PEG), which is known to be biocompatible [23]. To investigate the influence of reactive groups, we use PEG chains exhibiting either amine (NH_2_–PEG) or carboxy groups (COOH–PEG). The different surface coatings result in positively charged (CTAB, ζ-potential approx. +50 mV) [24], neutral (NH_2_–PEG, ζ-potential approx. 0 mV), and negatively charged (COOH–PEG, ζ-potential approx. −20 mV) nanoparticles, respectively. The ζ-potentials obtained from gel electrophoresis for PEG particles can be found in the Supporting Information of [20].

#### Live cell imaging

**Figure 1:**
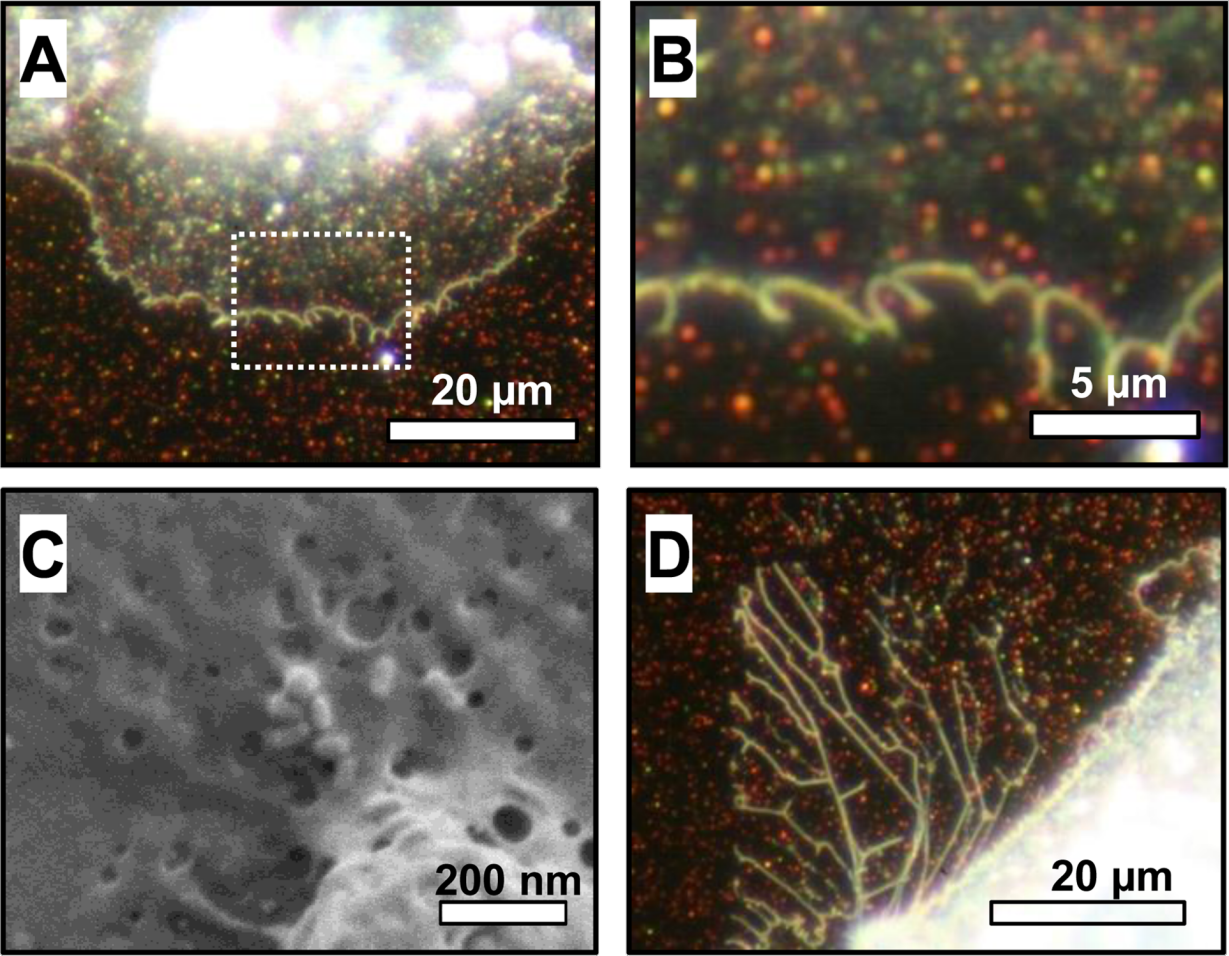
Growth of epithelial cells on a gold nanoparticle-decorated substrate. (A) Optical dark field microscopy detects light scattered by the sample. Gold nanoparticles appear as bright spots with colors corresponding to the plasmon frequency of the particle. The dense inner cell region with the strongly scattering cytoplasm appears bright white. The spread cell membrane is thin and translucent allowing observation of the nanoparticles underneath. (B) Magnified view of the nanoparticles covered with the membrane as marked in (A). (C) Scanning electron microscopy image of gold nanoparticles underneath the thin cell membrane. The membrane tightly covers the nanoparticles. (D) Optical dark field microscopy image of an area where the cell membrane has retracted. There are no irregularities or voids in the nanoparticle pattern, indicating that no nanoparticles were displaced. After retraction, a filamentous residue remains attached to the nanorod-decorated substrate. Since these filaments occur in the control samples of bare substrates as well, this behavior does not necessarily indicate an interaction of cell membrane and nanorods.

In order to investigate how single MDCK II cells adhere and grow on nanoparticle-decorated substrates, live cell imaging using optical dark field microscopy was performed. For basolateral exposure of nanoparticles, single gold nanorods were immobilized on the glass bottom of a petri dish with a growth area of 3.5 cm^2^ by the addition of salt solution. The salt solution screens the surface charges, which stabilize the nanoparticle suspension, leading to non-specific, random adsorption of nanoparticles onto the substrate. Once the nanoparticles are near the glass surface, they remain attached by van der Waals forces even in the absence of salt. Optical dark field microscopy was used to quantify the nanoparticle density on the substrate. In this technique, only scattered light from the sample is detected. Although the resolution is not powerful enough to image the actual particle shape, the gold nanorods scatter the light so efficiently (due to their plasmonic property) that the particles appear as bright spots on a dark background [1,4]. The spot size is more than 20 times larger in diameter than the actual size of the nanoparticles and the spot color is the plasmon frequency. This correlates to the particle shape: green spots are correlated with single gold nanospheres, while red spots correlate to individual gold nanorods. When the aggregation of two or more particles occurs, an orange or white spot appears. From the optical dark field microscopy images, we assume a particle density of 1–5 particles/µm^2^ on the substrate. To this nanoparticle-decorated substrate, a low number of about 6000 cells was added to ensure seeding of single cells. After four hours of incubation, the samples were investigated by optical dark field microscopy using a custom-built setup for live cell imaging. This setup consists of a conventional light microscope equipped with an acrylic glass compartment, which maintains culture conditions of 5% CO_2_ atmosphere at 37 °C. We observe the spreading of cells on the substrate for all samples regardless of nanoparticle coating. The inner cell region containing the nucleus, mitochondria, and dense cytoplasm scatters light so efficiently that it appears white and opaque (Figure 1A). In contrast, the spread cortical membrane is so thin and translucent that the scattered light from the particles below the membrane can pass through and is visible (Figure 1B: cortical membrane of Figure 1A enlarged). The membrane tightly covers the particles, which is verified by a scanning electron microscopy image in Figure 1C. When the cell membrane retracts, a filamentous residue remains (Figure 1D). However, this residue emerges in control samples without nanoparticle patterning as well (Supporting Information File 1, Figure S1) and corresponds to retracting filopodia [25]. Therefore, no specific anchoring of the membrane to the nanoparticles seems to be necessary for the occurrence of these filamentous membrane structures upon cell retraction. In addition to the filamentous residue, an even pattern of particles without irregular holes or clefts is maintained after retraction of the membrane. This indicates that there is no obvious removal of nanoparticles (Figure 1D). In a control experiment specifically testing for nanoparticle removal by the cell, cells were incubated on particles for seven days and the confluent cell layer was removed by trypsination. A nanoparticle pattern which was not affected by cell growth was observed, indicating that no nanoparticles were removed (Supporting Information File 1, Figure S2).

#### Growth behavior

**Figure 2:**
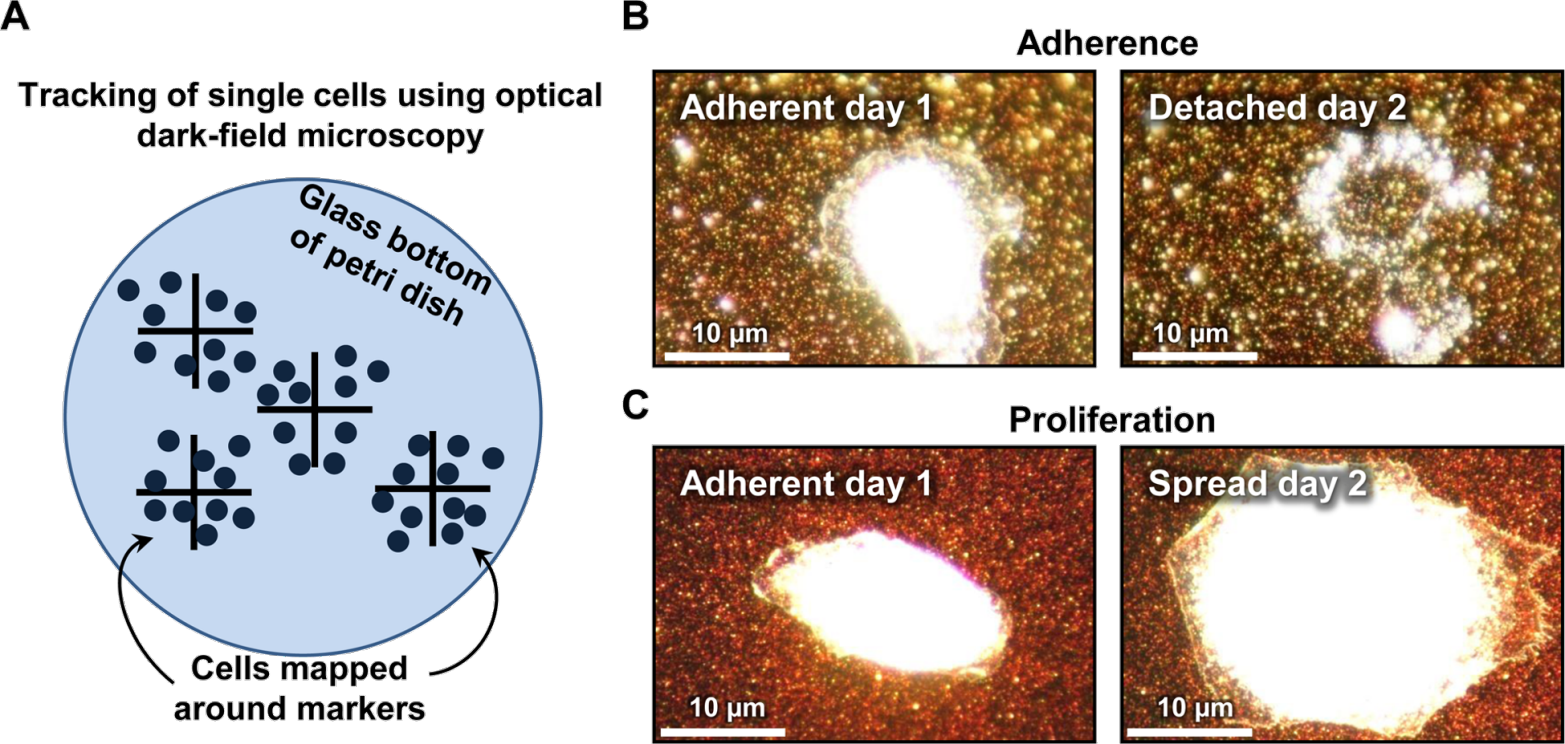
Live cell imaging. (A) Scratches into the glass were used as position markers in the glass bottom of a petri dish. After seeding, the cells were mapped around the markers and imaged daily over a period of three days. (B) Representative example for an adherent cell at day 1 shown on the left side, which disappeared at day 2, leaving behind the cell debris shown on the right side. (C) Representative example for an adherent cell at day 1 shown on the left side, which has considerably expanded its spreading area at day 2, indicating an active proliferation shown on the right side.

In order to characterize the growth behavior of epithelial cells on gold nanorod-decorated substrates, 50 spread cells on the substrate were chosen after 4 h of incubation. These cells were investigated on a daily basis over a period of three days. By using a mapping system of crosses scratched onto the substrate, we can easily track the same 50 cells chosen in the beginning of the experiments (Figure 2A). After tracking, the growth behavior of the cells was monitored regarding two aspects: by their adhesion and by their spreading. Herein, we assess the adhesion as an indicator for viability since the cells detach during apoptosis. Furthermore, an adherent cell, which has increased its spreading area, is considered to show an active proliferation. Figure 2B shows a typical example of an adherent cell at day one, which has detached at day two, leaving behind the cell debris. From the percentage of adherent cells, we rate their proliferation behavior shown by an active increase in spreading area. Figure 2C shows a representative image of a cell that is alive at day one and which has considerably expanded its spreading area at day two. This example is chosen as a representative of the average behavior in increase of the surface coverage with individual cells showing a smaller or even larger expansion compared to the presented cell.

#### Adherence

**Figure 3:**
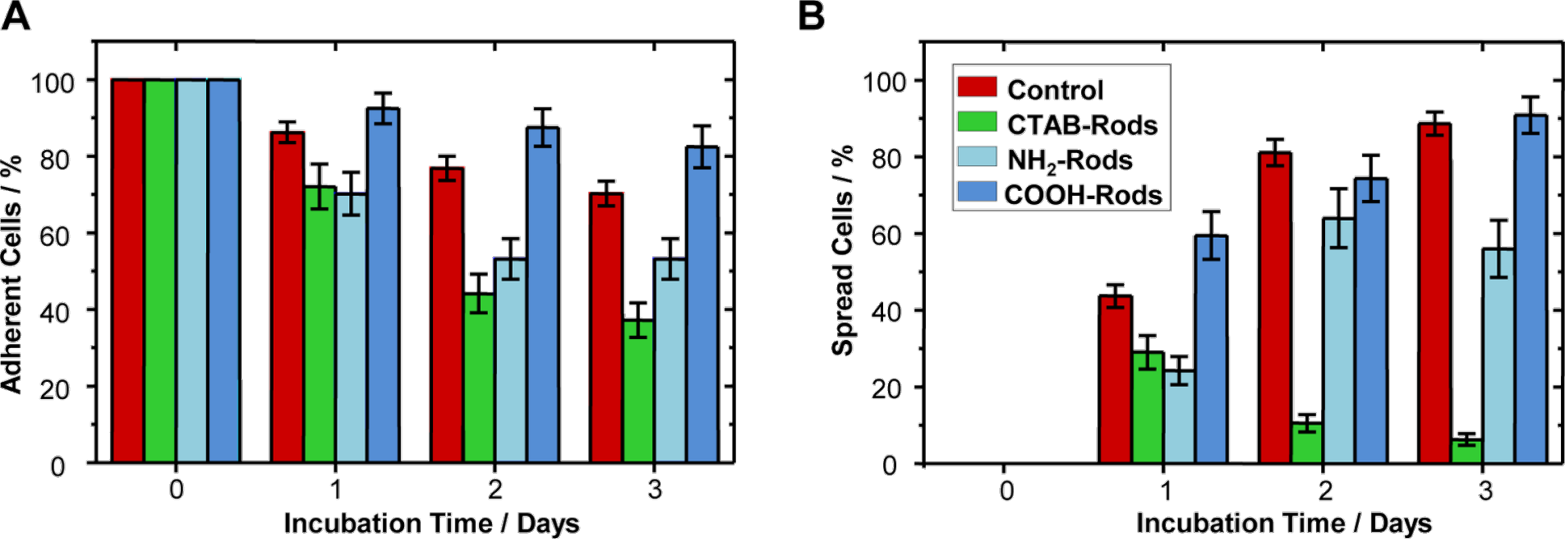
Adherence and proliferation of cells grown on nanoparticle-decorated substrates indicated by cell adhesion and spreading on the substrate. (A) Compared to the untreated control consisting of cells growing on a bare glass slide (red), the adherence of cells on CTAB nanorods (green) and NH_2_–PEG nanorods (light blue) is reduced by 45% and 30%, respectively, while adherence appears unaffected for COOH–PEG rods (blue). Note that in the control sample a few cells detached, since not every single seeded cell properly develops after initial attachment to the substrate. (B) Results indicating that over time, more and more cells increase their spreading area in the untreated control sample (cells growing on bare glass slide (red)). The same behavior was observed for cells growing on COOH–PEG nanorods (blue). In the presence of NH_2_–PEG nanorods (light blue), proliferation was reduced by 40% after 3 days, whereas proliferation of the tracked cells practically stopped within 3 days for CTAB rods (reduction compared to untreated control by 95%) (green). The data are presented as median and standard error of the mean.

The adherence of cells was indicated by the amount of adherent cells normalized to the starting number of cells chosen in the beginning of the experiment. In order to identify the number of adherent cells, each of the chosen cells was imaged. In these images, only the number of adherent cells present from the beginning of the experiment was counted. The percentage of adherent cells with respect to the number of starting cells for the different nanorod functionalizations is displayed in Figure 3A. At the start of the experiment, only adherent cells are chosen for all samples. The control sample shows the behavior of cells grown on a bare, nanoparticle-free substrate. Here, a small fraction of initially seeded cells detaches, since not every single cell properly develops after attachment to the substrate. A reduction by 45% (as compared to the untreated control) in the number of cells initially adherent resulted for the sample with a CTAB nanorods-patterned substrate. A reduction in adherence by 30% as compared to the untreated control results from the cell growth on the NH_2_–PEG nanorod-decorated substrate, whereas immobilization of COOH–PEG nanorods does not have an influence.

#### Proliferation

The increase in the spreading area of an adherent cell is interpreted as a sign of active proliferation. In order to quantify the proliferation, we inspect the adherent cells with respect to the increase in area covered by the membrane. For the analysis, neither the exact increase in the spreading area nor the actual number of adherent cells is quantified, but rather a visual evaluation regarding an obvious expansion (or not) is performed. Hence, the spreading variability among the cells is neglected and the extent of proliferation is not quantified. After inspection of each single cell, the number of cells showing an increase in spreading area is normalized to the number of adherent cells for the given day of incubation. These results are shown in Figure 3B. On the starting day, all chosen cells are adherent and the spreading area is recorded as a reference for comparison to the next day. Unaffected cells growing on a bare substrate as a control sample show an increasing fraction of adherent cells enlarging their spreading area over time. In contrast, the fraction of spreading cells growing on a CTAB nanorod-patterned substrate decreases more and more until proliferation nearly stops after three days of incubation. In the case of a NH_2_–PEG nanorod-patterned substrate, more and more cells spread between day one and two, but then the ratio of spreading cells among the living cells stagnates. For the COOH–PEG nanorods, we observe a similar proliferation behavior as for cells growing on the bare substrate, which is an increasing number of spreading cells.

#### Micromotility

**Figure 4:**
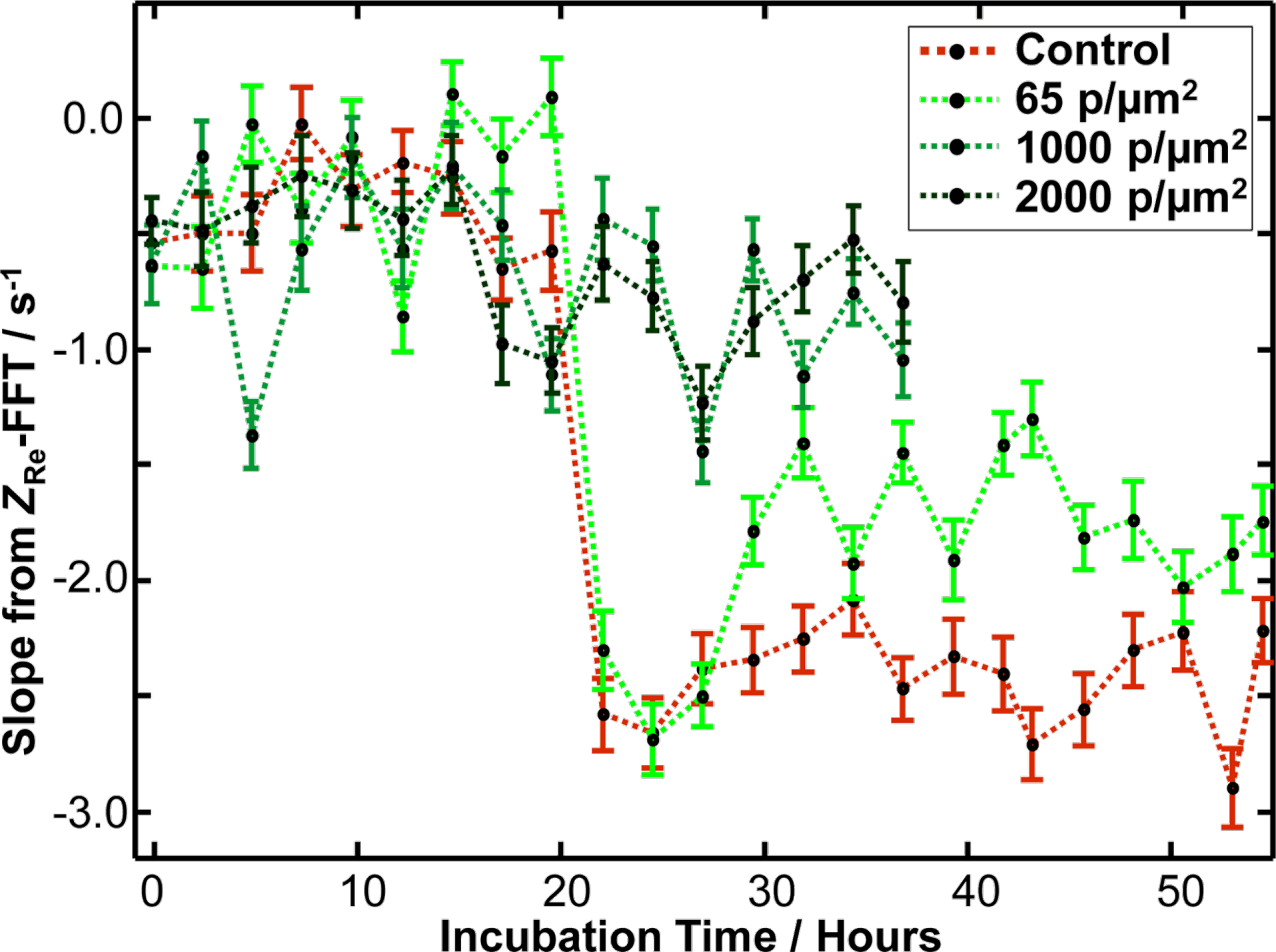
Slopes extracted from linear power spectral density regression in the low frequency regime of power spectra originating from *Z*_Re@4kHz_ impedance real part fluctuations for MDCK II cells seeded on CTAB nanorods with given densities at *t* = 20 h after nanoparticle seeding (green). Results for the untreated control consisting of cells growing on a bare substrate are displayed in red. Error bars indicate the standard deviation of the linear regression.

In addition to live cell imaging, a biocompatibility test was performed based on detecting the cell shape fluctuations of subconfluent cells cultured on small gold electrodes of 250 μm diameter, the so-called micromotion assay [18–19]. Electric cell–substrate impedance sensing (ECIS) is an electrochemical, non-invasive biosensor, which permits the monitoring of morphological changes of living cells acting as dielectric bodies in real time [26–27]. The method measures the complex impedance, *Z*, of a small working electrode and a larger counter electrode (Supporting Information File 1, Figure S3). The impedance spectrum of an uncovered ECIS electrode can be best described by an ohmic resistor *R*_bulk_ in series with a constant phase element (CPE) accounting for the capacitive properties of the fractal electrodes (Supporting Information File 1, Figure S4). Adhesion followed by spreading and eventually the formation of a confluent cell monolayer limits the current flow due to the insulating properties of cells and therefore produces a corresponding increase in *Z* as a function of applied AC frequency. In the high-frequency regime (ω > 10 kHz), the complex impedance is dominated by the capacitance of the cell membrane, *C*_m_ [28], while at lower frequencies the impedance is mainly determined by the current flow through intercellular gaps captured by an ohmic resistance *R*_b_. This resistance is a quantitative measure of the barrier properties of cell–cell contacts, that is, tight junctions. At even lower frequencies, the current flow between the cell and substrate dominates the impedance response. This contribution is represented by the parameter α_ECIS_, which is inversely proportional to the square root of the cell–substrate distance. A typical spectrum with a corresponding equivalent circuit of a cell-covered electrode is shown in Supporting Information File 1, Figure S4. Time-resolved measurements of *Z* at a fixed frequency, for instance a frequency at which the impedance is largely influenced by the parameter *α*_ECIS_, allows for the observation of the micromotion of cells. This micromotion therefore occurs due to collective changes in the cell–substrate distance [29]. The time series of impedance fluctuations is subject to a fast Fourier transformation (FFT) showing a power law behavior (*|Z*(ω)*| ≈* ω**^−^**^β^) with an exponent, β > 2, indicative of fractional Brownian motion, that is, long memory behavior (long correlation times) [30]. As only the CTAB nanorods display a significant impact on cell adherence and proliferation, we focus on these particles in the micromotion assay. In the assay, CTAB nanorods with mean particle densities from 65 to 2,000 particles/µm² were immobilized on the substrate and a baseline signal was collected for 20 h with a bare substrate serving as control. Afterwards, 300,000 cells were added to each well with an area of 0.8 cm^2^ (enough that a cell monolayer forms within a few hours) while recording the response. The results are presented in Figure 4. For the untreated control sample consisting of biologically active cells, a slope of −2.5 was found, as expected for living cells. In the case of CTAB nanorod-decorated substrates, mean particle densities of 1,000 to 2,000 particles/µm^2^ after cell addition result in mean slopes of −1.0. This result is comparable to the FFT slopes of electrodes immersed in culture medium (although cells adhere and spread, as judged optically) indicating the absence of migration-related fluctuations. The cells added to the substrate with a mean CTAB nanorod density of 65 particles/µm^2^ have the same fluctuation slope of −2.5 for the first 10 h of incubation as the untreated control cells resembling full viability. The slope then decreases to −1.6 after 35 h of incubation. Given a slope of −1.0 for the reference for inactive cells and a slope of −2.5 for active cells, a slope of −1.6 corresponds to micromotility, which is decreased by 60% compared to biologically active cells. This is in good accordance with the optical adherence and proliferation assay based on live cell imaging presented above.

## Discussion

In order to investigate cell growth behavior on glass substrates decorated with gold nanorods, living epithelial cells (MDCK II) were monitored using optical dark field microscopy. No pronounced cell migration away from the initial adhesion area or nanoparticle removal from the substrate was observed in contrast to previous reports on 3T3 fibroblast cells [15] or prostate carcinoma (PC3) and human dermal fibroblast (HDF) cells [14]. However, in this work, gold nanorods were purposely immobilized to the substrate using a salt solution, resulting in an attachment by van der Waals forces. This attachment could obviously not be reversed by the cells.

We investigated three different stabilizing agents present on the particle surface regarding their impact on the cells compared to growth on bare substrates, since the particle bound molecules (stabilizing agents) are expected to promote diverse interactions with the cell membrane [21–22]. One coating was cetyltrimethylammonium bromide (CTAB), which is a relatively cytotoxic cationic surfactant [11] used for particle synthesis. These CTAB molecules can be replaced by the inert polymer poly(ethylene glycol) (PEG) known for its biocompatibility [23]. Using PEG chains exhibiting either amine (NH_2_–PEG) or carboxy groups (COOH–PEG), the influence of exposed reactive groups was investigated. A strong influence on the cell adhesion as well for the spreading by CTAB nanorods was observed, with only a minor effect by NH_2_–PEG nanorods. No difference was observed for COOH–PEG nanorods as compared to the untreated control sample. Since there is at least one sample showing no impact (the COOH–PEG nanorods), we exclude negative effects on cell growth due to the act of decorating of the substrate itself. Therefore, the stabilizing agent, which comes into direct contact with the cell membrane, causes observable effects on viability and proliferation. Among the investigated stabilizers, CTAB is known to be cytotoxic [11] causing complexation of nucleic acids as well as protein/polysaccharide aggregation. These cytotoxic effects of CTAB clearly emerge in the proliferation, since after three days of incubation, nearly all cells stopped spreading while about 40% of the starting cells remained adherent. Here, cell adherence alone was not an adequate marker of cell viability, because due to the complexation properties of CTAB, the outer cell structure might have been fixed by the surfactant, which preserved the shape, while proliferation was prohibited. In order to have a reliable measure on viability, a staining test for live/dead should be performed. This was not performed since our results already indicated that CTAB nanorods are not suitable for live cell applications due to their impact on the native cell behavior. However, although the proliferation behavior of the tracked cells was poor, active cell division took place in the sample, which resulted in a confluent cell layer after seven days of incubation. In the micromotility assay, we found an impact of nanoparticle density (the number of particles available for one cell) on the cell behavior. Since CTAB nanorods are covered with a bilayer of the surfactant [31], the amount of CTAB exposed to the cells correlates with the particle density inducing cytotoxicity. For the live cell imaging assay, we replaced the cell medium for imaging and culturing, possibly reducing or eventually removing the CTAB bilayer during incubation, which could have resulted in a smaller impact on cells in a later stage of the experiment.

Since CTAB nanorods are positively charged [24], they are able to interact electrostatically with negatively charged membrane proteins, which are directly or indirectly linked to the actin filament of filopodia [22]. This interaction could have hindered cell migration, having an impact on the viability and proliferation of the cells. Over prolonged incubation time, a masking or replacement of CTAB by unspecific attachment of serum proteins from the culture medium could have taken place, reducing the positive ζ-potential significantly, even reversing to it to a negative ζ-potential [32]. Regardless, cells were able to attach to serum-coated nanoparticles via specific receptors. In any case, the interaction could hinder cell migration and impair cell growth. Since we observed no significant nanoparticle removal from the substrate, it is assumed that the interaction between the cell membrane and the nanoparticles was not strong enough to overcome the van der Waals forces keeping the particles attached to the substrate.

The other stabilizer investigated, PEG, is considered to be biocompatible [23]. This biocompatibility explains the observed similarity in cell growth of the untreated control sample and the sample decorated with COOH–PEG nanorods. In contrast, the presence of NH_2_–PEG nanorods reduced adherence by 30% and proliferation by 40%, as compared to cells growing on a bare glass substrate, which is inconsistent with the PEG biocompatibility itself. The antifouling properties of PEG prevent the efficient adsorption of serum proteins to the nanoparticles [33]. We attribute the difference between the functional PEG end groups to their different electrostatic attachment properties. The cell membrane is, on average, negatively charged. Electrostatic interaction with negatively charged COOH–PEG nanorods should therefore be repulsive, comparable to the bare glass substrates. In contrast, amines are known to interact with the negatively charged cell membrane. It is therefore likely that an anchoring of the membrane to the NH_2_–PEG nanorods impaired the growth behavior. However, any interaction between the nanorods and the membrane was not strong enough for removal of the immobilized particles from the substrate as there were no obvious irregularities in the nanoparticle-patterned substrate after retraction of the cell membrane.

We believe that the stabilizing agents CTAB and NH_2_–PEG promote contact of the nanoparticles to the cell membrane. We were not able to elucidate the specific type of interaction, but electrostatic attraction, receptor response to unspecifically attached serum proteins on the nanoparticle surface, and direct chemical binding are possible candidates. For an internalization of particles into the cell, the contact strength between the particle and the membrane must overcome the van der Waals forces, keeping the particles immobilized on the substrate. In regions where the cell membrane retracted, particles are left behind and a filamentous residue remained. However, these filaments were not an indicator for an irreversible nanoparticle membrane interaction since they were present in untreated control samples as well. Therefore, we conclude the contact strength between particles and membrane was weaker than the van der Waals forces between immobilized particles and substrate in our case. In contrast, the cells were able to remove larger particles from substrates in other studies [14]. In the light of these conflicting results, further studies into the minimal adhesion strength nanoparticles on substrate require in order to prevent their removal by cells seem to be necessary.

In previous studies, the cytotoxic impact of apical exposure of the same functionalized nanoparticles to the same epithelial cell line (MDCK II) was presented [18] and the cellular uptake was quantified [20]. It was found that about 20% of apical applied CTAB nanorods enter the cell, whereas only a fraction of a percent of PEG nanorods (regardless of end group) were internalized. We attributed the differences in uptake to the surface charge of the particles favoring or disfavoring electrostatic interaction with the negatively charged membrane [21–22], and to the sedimentation behavior of the particles which is related to their colloidal stability under physiological conditions [33]. The uptake was likely caused by non-specific endocytosis or macropinocytosis. Therefore, we expected an even stronger uptake of basolaterally applied particles, since MDCK II cells exhibit caveolae on the basolateral side [17]. However, the immobilization of the particles on the substrate could not be overcome by the cell (see discussion above). Hence, we conclude that there is no significant basolateral nanoparticle uptake from patterned substrates, which implies that nanoparticle removal from implants by epithelial cells is negligible.

Concerning cytotoxicity, apical exposure of CTAB nanorods reduced mitochondrial activity compared to untreated cells, whereas PEG nanorods showed no impact, regardless of end group [20]. Taking a closer look at the cytoskeleton of cells after apical exposure to CTAB rods, we observed gaps between adjacent cells where the cortical ring previously existed and unusual aggregates of actin in the cytosol. Furthermore, β-tubulin was redistributed as monomers towards the cell periphery, and the cells covered a smaller area compared to untreated cells [18]. Apical addition of PEG particles did not induce visible changes in the cytoskeleton [18].

The basolateral nanoparticle application used in the present study showed interesting deviations from the two previous studies [18,20]. CTAB nanorods induced a reduction in proliferation compared to untreated cells, similar to apical addition, but remarkably the particles were not internalized. In case of NH_2_–PEG nanorods, basolateral exposure resulted in a reduced viability compared to untreated cells without observable particle uptake. For COOH–PEG nanorods, apical as well as basolateral nanoparticle contact showed the same findings as untreated cells. Therefore, basolateral presence of nanoparticles has an influence on cell growth behavior depending on the stabilizing agent exposed to the cell membrane. The influence is independent from internalization. Hence, applications involving nanoparticle patterning of implants should also consider any stabilizing agents with respect to cellular interaction.

## Conclusion

Gold nanoparticles scatter and absorb light strongly, which makes them amenable to biomedical applications. However, unintended impact on biological tissue should be carefully considered. Since studies on nanoparticle–cell interactions thus far were focused solely on the apical application of particles to adherent cells, we tracked the growth behavior of epithelial cells having variously functionalized particles basolaterally exposed, which indicates, for example, cellular response to particle-treated implants. We found an impaired cell growth correlated to the cytotoxicity of the surface bound surfactant. In the case of the presence of a biocompatible polymer on the nanoparticles, we observed no effect on cell growth for the functional end group –COOH whereas the functional end group –NH_2_ reduced adherence and proliferation compared to cells growing on a bare glass substrate. This was initially unexpected given the cytotoxicological properties of the polymer. We conclude that the impact of gold nanorods on epithelial cells is not simply related to the cytotoxicological properties of the surface bound moieties. We assume that cellular contact with NH_2_–PEG particles results in a disturbed growth process. This interaction should be characterized and studied in more detail to understand the nanomaterial characteristics leading to an unintended influence. However, the impact of basolateral exposure of gold nanorods on epithelial cells depends critically on the exposed chemical moiety in contact with the cell membrane and has to be evaluated to assess the effect of patterned implants.

## Experimental

Deionized water from a Millipore system (>18 MΩ, Milli-Q) was used in all experiments. Suppliers of chemicals are given in the Supporting Information File 1.

### Particle synthesis

Gold nanorods were synthesized according to the seeded growth method published by Nikoobakht [34] as presented in [20]. In a first step, the seeds were prepared by adding ice-cold sodium borohydride (NaBH_4_, 0.6 mL, 0.010 M) to a solution of cetyltrimethylammonium bromide (CTAB, 10 mL, 0.1 M) containing tetrachloroauric acid (HAuCl_4_, 50 µL, 0.1 M) under vigorous shaking. In a second step, the nanorods were prepared by adding the seed solution (12 µL) to a growth solution consisting of HAuCl_4_ (75 µL, 0.1 M), CTAB (10 mL, 0.1 M), silver nitrate (AgNO_3_, 7 µL, 0.04 M), and ascorbic acid (AA, 105 µL, 0.0788 M). Shortly before the experiment, the nanoparticles were washed in two centrifugation steps with water to remove unbound CTAB.

### Particle characterization

Particles were characterized in the same manner as described in [20]. The nanoparticle size was determined using transmission electron microscopy (TEM). In order to prepare the samples, a drop of a solution (about 10 µL) with an approximated concentration of 6 × 10^13^ particles/mL was dropped on a carbon-coated copper grid (Plano) placed on filter paper. After drying, images were taken on a Philips EM420 using an operating voltage of 120 kV. The mean nanoparticle size and standard deviation was taken for at least 270 particles. A representative TEM images and size distribution histograms are given in the Supporting Information of [20]. We determined a length of 37.8 ± 6.5 nm and a width of 17.2 ± 2.9 nm for the nanorods.

### Particle concentration

The concentration of the nanoparticle solution was calculated from the optical extinction value at 400 nm, as presented in [20]. The molar extinction coefficient for the nanorods was calculated using the Mie–Gans theory in the quasi-static approximation [35] yielding 1.1 × 10^9^ L mol^−1^ cm^−1^. Hence, the stock solution had a concentration of 6.2 × 10^11^ particles/mL corresponding to 88.9 µg Au/mL.

### Particle functionalization

In order to replace CTAB, various functionalized poly(ethylene glycol) thiols (X–PEG–SH, where X = COOH, NH_2_, CH_3_O; MW = 5000 Da) were grafted onto the gold surface [36] in the same manner as [20]. For this purpose, we incubated the nanoparticle pellet overnight with 100 µL of an aqueous 2 mM mixture of 75% NH_2_–PEG–SH and 25% CH_3_O–PEG–SH (NH_2_–PEG particles) or 75% COOH–PEG–SH and 25% CH_3_O–PEG–SH (COOH–PEG particles), respectively. The next day, excess PEG was removed by centrifugation. The success of the PEGylation was tested by gel electrophoresis, which also reveals the surface charge of the particles (Supporting Information of [20]).

### Cell culture

In our studies, we used epithelial MDCK (type II) cells and performed cell culture as described in [20]. Cells were cultured in Earle’s minimum essential medium supplemented with glutamine (4 mM), penicillin and streptomycin (100 µg/mL for both), fetal calf serum (10% v/v) and stored in an incubator with 5% CO_2_ atmosphere at 37 °C (HERA cell 150, Heraeus). Subculture was performed weekly after cells reached confluence. After the medium was removed, the cell monolayer was washed twice with phosphate-buffered saline (PBS, 4 mL) without magnesium and calcium ions and incubated with the chelating agent ethylenediaminetetraacetic acid (EDTA, 2 mL) for 10 min in the incubator. Then, EDTA was removed and cells were detached from the substrate by incubation with trypsin/EDTA (1 mL) for 10 min in the incubator. Trypsination was stopped by addition of medium (10 mL), which was removed afterward by centrifugation at 110 g for 10 min. Cells were resuspended in fresh medium (10 mL) and seeded into a new culture flask at a ratio of 1:10 [37].

### Cell growth on patterned substrates

Markers for cell tracking shown in Figure 2A were scratched with a diamond-tipped pencil into the glass bottom of a petri dish. Afterwards, the petri dish was treated with oxygen plasma for 45 s to render the surface hydrophilic. For sample preparation, 100 μL of 1 M sodium chloride and 100 μL of the particle solution or 100 μL of water in case of the untreated control sample were put into the petri dish. After incubation for 30 min, the samples were rinsed three times with 3 mL of water to remove any particles that were not immobilized. Water was replaced by 500 μL of culture medium filtered through a sterile syringe filter with a 200 nm pore size to minimize the risk of large scattering centers, which could add background in optical dark field microscopy measurements. Finally, 10 μL of the cell suspension (≈6000 cells) were seeded and samples were kept under cell culture conditions.

### Live cell imaging

Optical dark field microscopy was performed after 4, 24, 48, and 72 h while maintaining 37 °C and a 5% CO_2_ environment. In order to improve contrast for imaging, the cell culture medium was replaced by a version without a phenol red pH indicator or any nutrients. As soon as imaging was finished, the medium was changed back to the full culture medium and the samples were kept in a cell culture incubator.

### Electric cell–substrate impedance sensing (ECIS)

ECIS measurements were performed according to [38]. A custom-built ECIS system was employed, consisting of a lock-in amplifier (SR830, SRS, Inc., Sunnyvale, CA), an internal oscillator, and a homemade multiplexer for automatic analog stepwise switching between connections for multiple wells on the commercially available sensor chip (8w1E, Applied biophysics, Troy, NY, USA). The chip consists of eight separate wells with an area of 0.8 cm², the substrate-integrated gold electrodes are 250 μm in diameter and share a large (7 × 46 mm^2^) common counter electrode. Here, a 1 V AC signal with a 1 MΩ series resistor is applied and in- and out-of-phase voltages are recorded at 4 kHz with a sampling of 550 points at 1 Hz. The voltages are proportional to real (resistance) and imaginary (capacitive reactance) parts of the complex impedance and modified by the motility of adherent cells onto the circular gold electrodes. We applied only 1 μA current amplitudes in order to be as noninvasive to the epithelial cells as possible. Noise analysis of time series of resistance fluctuations was carried out by fast Fourier transformation (FFT). Linear fitting was performed in the regime from 10^−0.5^ to 10**^−^**^1.5^ Hz, as described by Giaever et al. [29], which corresponds to slopes ranging from **−**2.1 to **−**3 s**^−^**^1^ for fully motile epithelial cells exhibiting 100% micromotion and 0 to **−**1 for bare electrodes as well as fixed cells immersed in buffer. The time-dependent slopes were recorded for various surface densities of CTAB nanorods ranging from 65 to 2,000 particles/µm^2^. After 20 h, 300,000 cells were added.

### Scanning electron microscopy (SEM)

The cells were grown for 4 h on 12 mm round coverslips decorated with a density of 1–5 particles/µm^2^ and afterwards fixed by incubation with 2.5% glutaraldehyde for 1h. The fixing agent was removed and the sample was rinsed three times with PBS. Afterwards the sample was immersed in 10%, 25%, 50%, 75%, and 95% ethanol for 30 min each. Finally, the sample was covered with 100 % ethanol overnight. After dehydration, the sample was dried in a nitrogen flow and coated with a 15 nm thick gold layer. Cells were examined with a Leo Supra 55VP SEM (Zeiss, Oberkochen, Germany) at a voltage of 200 kV.

## Supporting Information

File 1 Materials, results of control experiments, principle of ECIS measurements.
