# Supplementary material for: Mammalian cell growth on gold nanoparticle-decorated substrates is influenced by the nanoparticle coating
Source: Beilstein J Nanotechnol. 2014 Dec 24;5:2479–88. doi: 10.3762/bjnano.5.257 (PMC4311695; doi:10.3762/bjnano.5.257)
Supplement: File 1 — Materials, results of control experiments, principle of ECIS measurements. [file Beilstein_J_Nanotechnol-05-2479-s001.pdf]

Supporting Information

for

Mammalian cell growth on gold nanoparticle-decorated substrates is influenced by the nanoparticle coating

Christina Rosman[1,§], Sebastien Pierrat[1,2], Marco Tarantola[3], David Schneider[4,5], Eva Sunnick[4], Andreas Janshoff[4], and Carsten Sönnichsen*[1]

Address: [1]Institute of Physical Chemistry, University of Mainz, Duesbergweg 10–14, 55128 Mainz, Germany, [2]Current address: Fraunhofer Institute for Microelectronic Circuits and Systems (IMS), Finkenstraße 61, 47057 Duisburg, Germany, [3]Laboratory for Fluid Dynamics, Pattern Formation and Biocomplexity, Max Planck Institute for Dynamics and Self-Organization (MPIDS), Am Fassberg 17, 37077 Göttingen, Germany, [4]Institute of Physical Chemistry, University of Göttingen, Tammannstraße 6, 37077 Göttingen, Germany, and [5]Current address: Max Planck Institute for Biology of Ageing, Joseph-Stelzmann-Straße 9b, 50931 Cologne, Germany

Email: Carsten Sönnichsen* - Soennichsen@uni-mainz.de

* Corresponding author

§ now: Christina Lambertz

Materials, results of control experiments, principle of ECIS measurements

Materials

Hydrogen tetrachloroaurate trihydrate, sodium borohydride, ascorbic acid, silver nitrate, cetyltrimethylammonium bromide, sodium chloride, glutaraldehyde, PBS, and ethanol were purchased from Sigma-Aldrich. Thiolated methoxy-polyethylene glycol, thiolated amine-polyethylene glycol, and thiolated carboxy-polyethylene glycol (MW = 5000 Da) were purchased from Iris Biotech. Earle's minimum essential medium, L-glutamine, penicillin, streptomycin, phosphate-buffered saline without magnesium and calcium ions, ethylenediaminetetraacetic acid, and trypsin 0.05% were purchased from Biochrom. Fetal calf serum was purchased from PAA. Sucrose was purchased from Fluka. Sterile, untreated petri dishes with glass bottoms (µ-dish, 35 mm) were purchased from Ibidi. Sterile Anotop 25 disposable syringe filters with a pore size of 200 nm were purchased from VWR.

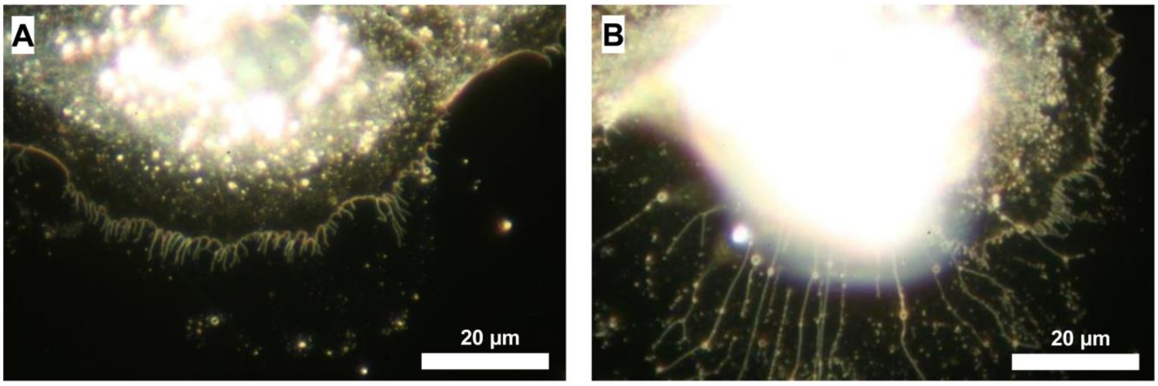

Figure S1: These images show examples of the filamentous residue on a bare glass substrate which remains after retraction of the cell membrane.

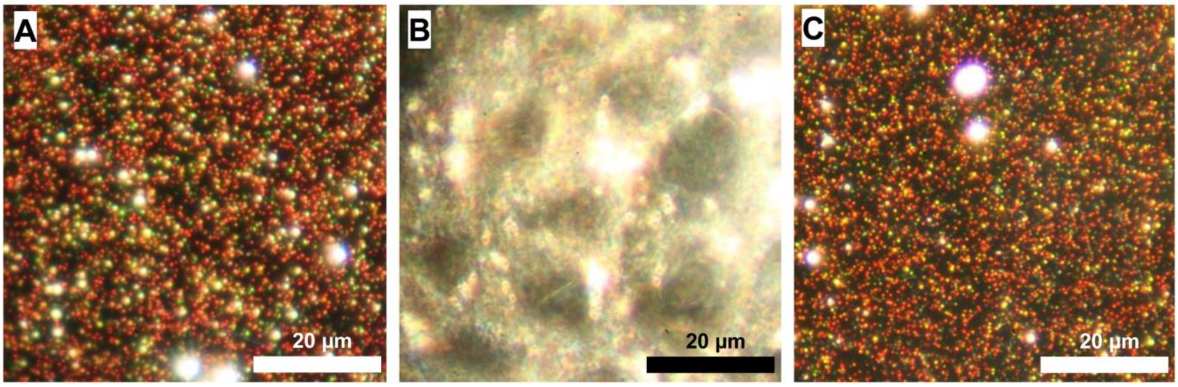

Figure S2: Experiment to test for nanoparticle uptake from the substrate by the adherent cells. (A) Substrate patterned with CTAB-nanorods. (B) After seven days of incubation, a monolayer of cells covers the substrate. (C) Cells were removed by trypsin. The substrate pattern before and after cell growth shows a comparable density. Hence, MDCK II cells do not internalize the particles to any remarkable extent.

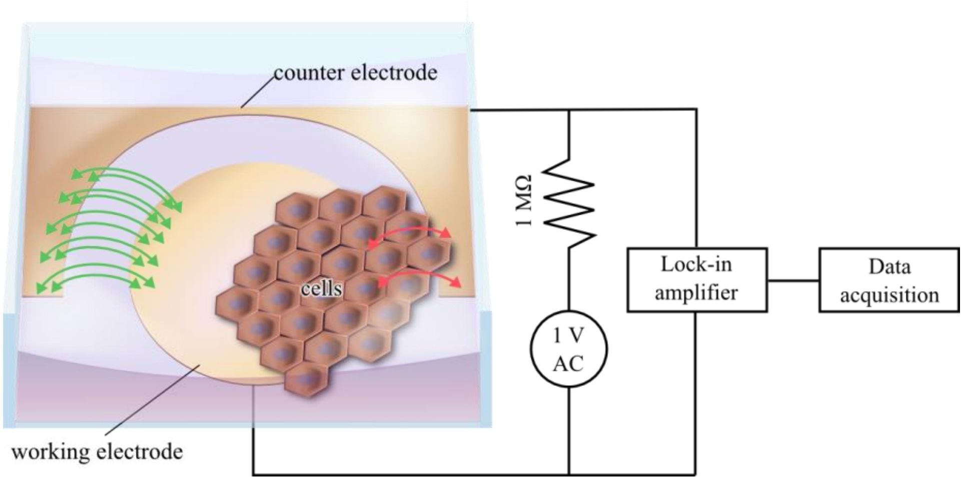

Figure S3: Scheme illustrating an ECIS setup consisting of a working electrode and a considerably larger counter electrode, both immersed in cell culture medium. An alternating current (AC, green arrows) is applied between both electrodes. Cells adhered to the electrode reduce current flow and the impedance increases accordingly (red arrows). A lock-in amplifier separates amplitude and phase of the AC current. This scheme was reprinted with permission from the PhD thesis "Dynamics and mechanics of adherent cells in the context of environmental cues" by Jan Rother, Goettingen (2014).

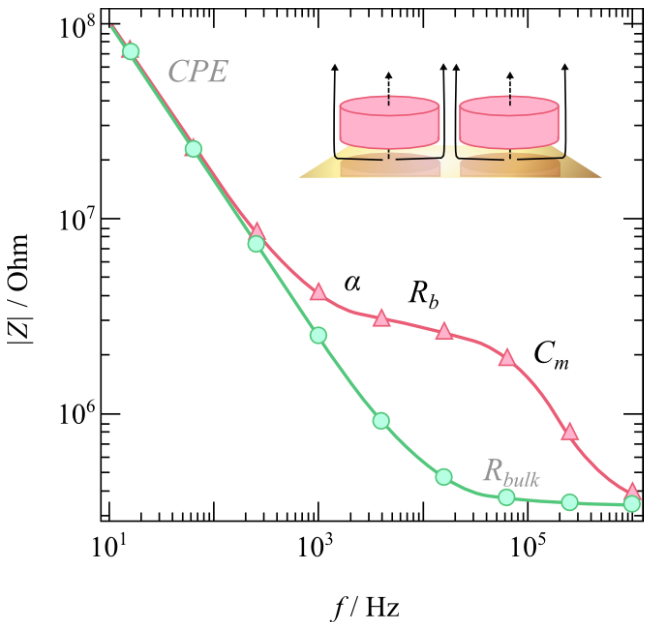

Figure S4: Typical impedance spectra of a cell-free (green) and a cell-covered (red) gold electrode. Inset shows the current flow (black arrows) on a cell-covered electrode (red discs). This scheme was reprinted with permission from the PhD thesis "Dynamics and mechanics of adherent cells in the context of environmental cues" by Jan Rother, Goettingen (2014).
